# Supplementary material for: Mechanisms governing the pioneering and redistribution capabilities of the non-classical pioneer PU.1
Source: Nat Commun. 2020 Jan 21;11:402. doi: 10.1038/s41467-019-13960-2 (PMC6972792; doi:10.1038/s41467-019-13960-2)
Supplement: Supplementary file 7 — Source data [file 41467_2019_13960_MOESM7_ESM.zip › Source_Data/FigureS2/FigureS2B_MotifScanOutput/KmeansCluster14/homerResults/motif19.similar.html]

motif19

## Information for motif19

G
C
A
T
A
C
T
G
C
G
T
A
A
G
T
C
A
C
G
T
A
T
G
C
C
G
T
A
  
Reverse Opposite:  

A
C
G
T
T
A
C
G
G
T
C
A
A
C
T
G
A
C
G
T
A
G
T
C
C
G
T
A
  

|  |  |
| --- | --- |
| p-value: | 1e-16 |
| log p-value: | -3.766e+01 |
| Information Content per bp: | 1.868 |
| Number of Target Sequences with motif | 139.0 |
| Percentage of Target Sequences with motif | 27.25% |
| Number of Background Sequences with motif | 5964.0 |
| Percentage of Background Sequences with motif | 13.17% |
| Average Position of motif in Targets | 99.3 +/- 57.2bp |
| Average Position of motif in Background | 99.1 +/- 69.6bp |
| Strand Bias (log2 ratio + to - strand density) | -0.2 |
| Multiplicity (# of sites on avg that occur together) | 1.16 |
| Motif File: | file (matrix) reverse opposite |

### Similar de novo motifs found

|  |  |  |  |  |  |  |  |
| --- | --- | --- | --- | --- | --- | --- | --- |
| Rank | Match Score | Redundant Motif | P-value | log P-value | % of Targets | % of Background | Motif file |
| 1 | 0.867 | T A G C A T G C C T G A C T A G C A G T T A C G G C T A A T G C G C A T A G T C G T C A A G T C C A G T | 1e-14 | -33.322125 | 6.67% | 1.19% | motif file (matrix) |
| 2 | 0.874 | T G C A A C G T C T A G C G T A A T G C G C A T A G T C C G T A A G C T A T C G T A C G | 1e-14 | -33.101697 | 4.31% | 0.42% | motif file (matrix) |
| 3 | 0.890 | C A G T T C G A G A C T T C A G C G T A A T G C A C G T T A G C G C T A | 1e-13 | -31.397347 | 17.84% | 7.50% | motif file (matrix) |
| 4 | 0.831 | C T G A A C G T A C T G C G T A A T G C C G A T A G T C C G T A A G C T A C T G | 1e-13 | -31.331637 | 4.51% | 0.52% | motif file (matrix) |
| 5 | 0.911 | C G A T C T A G G T C A T A C G A C G T A T G C C G T A A G C T | 1e-12 | -28.753140 | 6.86% | 1.49% | motif file (matrix) |
